# The impact of EAES Fellowship Programme: a five-year review and evaluation

**DOI:** 10.1007/s00464-021-08525-8

**Published:** 2021-06-08

**Authors:** Alice Tsai, Marek Soltes, Dusan Lesko, Michel Adamina, Pedrag Andrejevic, Milos Bjelovic, Kenneth Campbell, Mark Coleman, Nicoló de Manzini, Samir Delibegovic, Wlodzimierz Majewski, Ewelina Malanowska, Lubomir Martinek, Peter Sedman, György Lázár, Konstantinos Tsalis, Dorin Popa

**Affiliations:** 1 Department of Surgery and Cancer, Imperial College London, London, UK; 2 1st Department of Surgery, University of Pavol Jozef Safarik, Kosice, Slovak Republic; 3 1st Department of Surgery, University Hospital of L.Pasteur, Kosice, Slovak Republic; 4 Department of Surgery, Cantonal Hospital of Winterthur, Winterthur, Switzerland; 5 Department of General Surgery, Mater Dei Hospital, Msida, Malta; 6 Department for Minimally Invasive Upper GI Surgery, University Hospital for Digestive Surgery, Belgrade, Serbia; 7 Department of Colorectal Surgery, Ninewells Hospital & Medical School, Dundee, UK; 8 Department of Colorectal Surgery, University Hospitals Plymouth NHS Trust, Plymouth, UK; 9 Director of Surgical Department, University of Trieste, Trieste, Italy; 10 Clinic for Surgery, University Clinical Center, Tuzla, Bosnia and Herzegovina; 11 Medical Simulation Centre, Pomeranian Medical University, Szczecin, Poland; 12 Department of Gynecology, Endocrinology and Gynecologic Oncology, Pomeranian Medical University, Szczecin, Poland; 13 Department of Surgery, Faculty of Medicine, University of Ostrava and University Hospital Ostrava, Ostrava, Czech Republic; 14 Department of General Surgery, Hull University Teaching Hospital NHS Trust, Cottingham, UK; 15 Department of Surgery, University of Szeged, Szeged, Hungary; 16 Aristotle University of Thessaloniki, Thessaloniki, Greece; 17 Department of General Surgery, Linköping University Hospital, 581 85 Linköping, Sweden

**Keywords:** Fellowship, Fellow, Education, Training, EAES, Minimally invasive

## Abstract

**Background:**

The European Association of Endoscopic Surgery (EAES) fellowship programme was established in 2014, allowing nine surgeons annually to obtain experience and skills in minimally invasive surgery (MIS) from specialist centres across the Europe and United States. It aligns with the strategic focus of EAES Education and Training Committee on enabling Learning Mobility opportunities. To assess the impact of the programme, a survey was conducted aiming to evaluate the experience and impact of the programme and receive feedback for improvements.

**Methods:**

A survey using a 5-point Likert scale was used to evaluate clinical, education and research experience. The impact on acquisition of new technical skills, change in clinical practice and ongoing collaboration with the host institute was assessed. The fellows selected between 2014 and 2018 were included. Ratings were analysed in percentage; thematic analysis was applied to the free-text feedbacks using qualitative analysis.

**Results:**

All the fellows had good access to observing in operating theatres and 70.6% were able to assist. 91.2% participated in educational activities and 23.5% were able to contribute through teaching. 44.1% participated in research activities and 41.2% became an author/co-author of a publication from the host. 97.1% of fellows stated that their operative competency had increased, 94.3% gained new surgical skills and 85.7% was able to introduce new techniques in their hospitals. 74.29% agreed that the clinical experience led to a change in their practices. The most commonly suggested improvements were setting realistic target in clinical and research areas, increasing fellowship duration, and maximising theatre assisting opportunities. Nevertheless, 100% of fellows would recommend the fellowship to their peers.

**Conclusion:**

EAES fellowship programme has shown a positive impact on acquiring and adopting new MIS techniques. To further refine the programme, an individualised approach should be adopted to set achievable learning objectives in clinical skills, education and research.

**Supplementary Information:**

The online version contains supplementary material available at 10.1007/s00464-021-08525-8.

The European Association of Endoscopic Surgery (EAES) fellowship programme was established in 2014 based on the needs and feedback of the EAES members. It is awarded to on average nine young surgeons annually and provides the network and financial support to obtain experience and skills in minimally invasive surgery (MIS) from specialist centres across the Europe and the United States. The EAES fellowship aligns with the vision of EAES Education and Training Committee including a strategic focus on enabling Learning Mobility opportunities.

Learning Mobility in surgery can be defined as transnational mobility for the acquisition of new skills and innovations, in which the application of international fellowship programmes has gained recognition over the last 30 years. The International Guest Scholarship (IGS) Programme of the American College of Surgeons (ACS) formalised in 1968, Moynihan Travelling Fellowship in 1937 and BJS Travelling Fellowship in 1979 of the Association of Surgeons of Great Britain and Ireland (ASGBI) are three of the most established travelling fellowships. The survey of IGS in 2003 showed direct positive effect on clinical care including learning of new techniques that were in turn practiced in the scholar’s department and change in clinical practice that led to improvements in their work [1] . Many described that new ideas for research were picked up and they began new studies in their own countries. A more recent survey of the American Pediatric Surgical Association (APSA) travel fellowship in 2020 evaluated the experience of 11 surgeons from low-and middle income countries participating in clinical and didactic activities through observership in the US. It demonstrated that the majority of the fellows had implemented important changes in their hospital’s health systems including research and quality initiatives to improve pediatric surgical care [2]. Understanding the benefits in clinical care, surgical advancement, and international collaborations, many organisations including surgical societies, technology industries and regulatory bodies now offer a range of opportunities for surgeons [3].

The purpose of EAES fellowship is to provide exposure to expertise in MIS that may not have been otherwise gained and for the Fellow to be involved in clinical, educational and research activities at the host institution. Since 2014, 42 fellows have been through the programme. In order to assess the impact of the programme over the 5 years, this study conducted a survey of these fellows to assess the experience gained, the impact to practice in their home countries and seek feedback for improvements.

## Materials and methods

A questionnaire (supplementary file 1) using a 5-point Likert scale was developed to evaluate the fellow’s clinical experience including participating in operating theatres, wards and outpatient activities as well as education and research experience including accessing local meetings and research studies. The impact of the fellowship was assessed through the rating of acquisition of new technical skills, change in clinical practice and ongoing collaboration with the host institute.

Selected descriptive data including the demographics and free-text feedbacks were also collected. Ethics approval was not required as this study was an evaluation and not answering a research question.

The fellowship winners between 2014 and 2018 were included; 2019 were excluded due to the disruption of programme as a result of Covid-19 pandemic. The survey was confidential and anonymous. It was distributed to all 42 fellows through an email invitation using SurveyMonkey®. The proportion of the 5 point Liker scale ratings was analysed in percentage; thematic analysis was applied to the free-text feedbacks where data points in short paragraph or phrases were coded using qualitative analysis software, NVivo12.

## Results

### Demographic

**Fig. 1:**
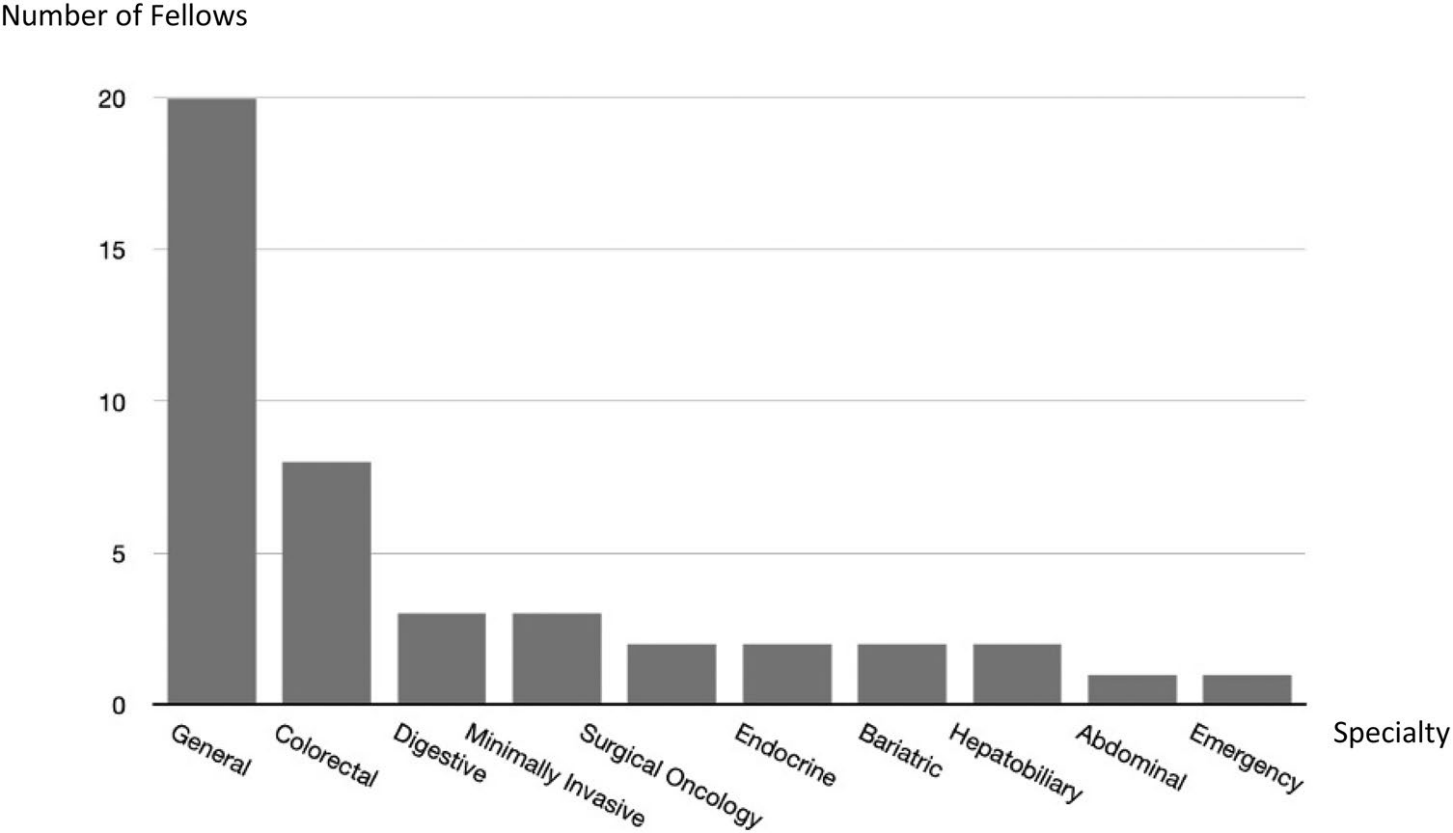
Surgical specialties declared by EAES fellows

**Fig. 2:**
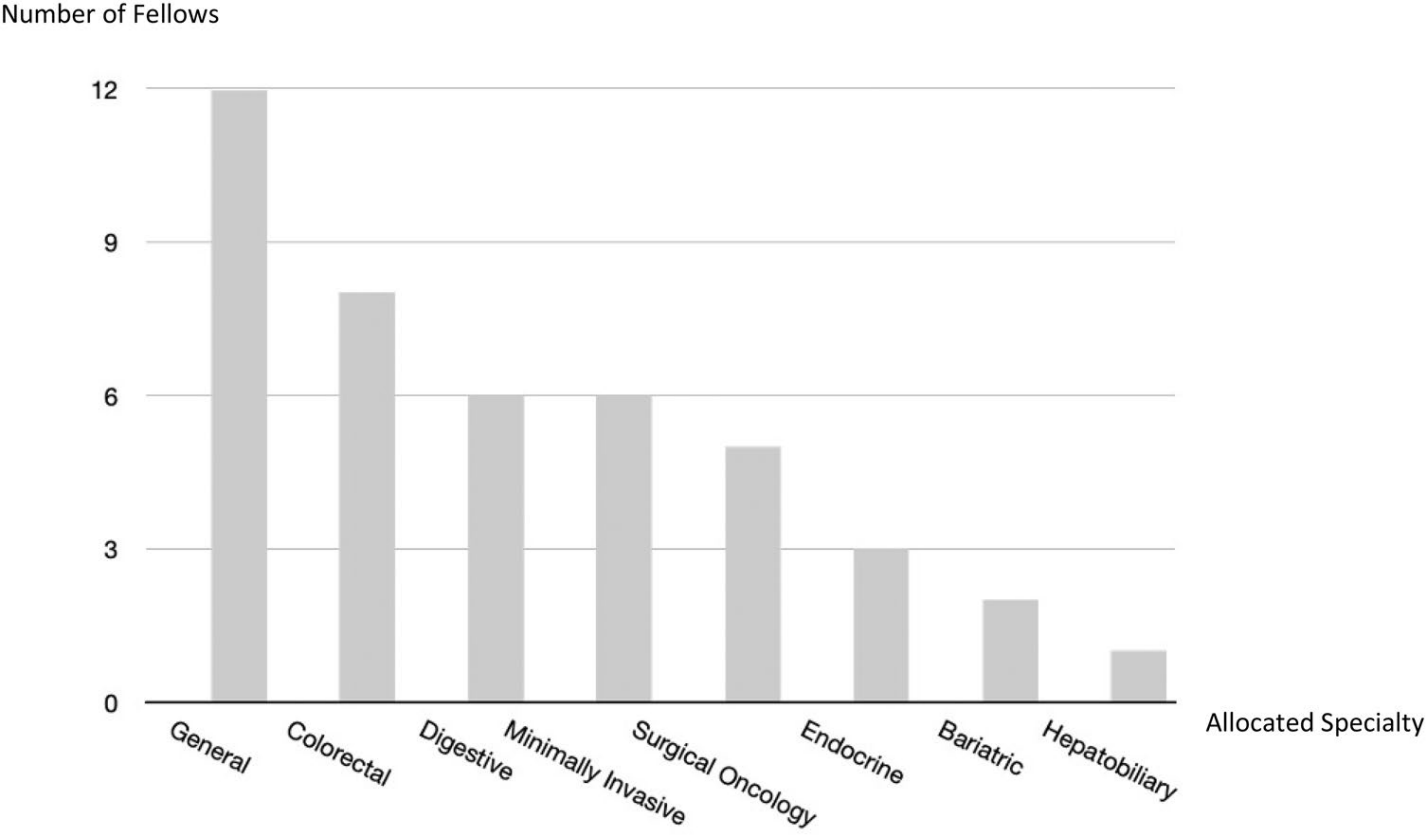
Surgical specialties that the fellows were allocated to during the fellowship

Forty-two fellows were approached and 35 (83%) participated in the survey; 31 fellows were consultant surgeons and 4 residents during the fellowship period. The average years of independent practice prior to the fellowship amongst the consultant surgeons was 5.7 (range 1–13 years). As for the surgical specialty(ies) of the participants, 20 declared in general surgery, 8 in colorectal, 3 in digestive, 3 minimally invasive and 2 endocrine; some surgeons declared more than one specialty (Fig. 1). During the fellowship period, participants may have placements with more than one specialty; 12 surgeons were allocated to colorectal surgery, 8 bariatric, 6 minimally invasive, 6 upper GI, 5 digestive, 3 general, 2 oncology and 1 hepatobiliary (Fig. 2).

### Clinical and operative exposure

All the fellows rated their access to attending and observing in the operating theatre as good (91.2% strongly agreed, 8.8% agree). 70.6% (55.9% strongly agree, 14.1% agree) were able to assist in operations; 14.7% strongly disagreed. 67.7% (41.2% strongly agree, 26.5% agree) had good access to clinical activities such as ward rounds and outpatient clinics and 64.7% (29.4% strongly agree, 35.3% agree) felt that they were able to participate and contribute to clinical care. Factors that contribute to experience in clinical exposures and operating theatres were identified; relevant paragraph and phrases were extracted as codes (Table 1). Positive experience in clinical and operative exposure was directly associated with positive attitudes of the hosts (4 codes), hands-on participation (2 codes) and expertise of the chief surgeons (2 codes).

unlimited access to operation room, approachability of faculty and residents were the best part

great support from the chief surgeon and his staff

Assisting Professor in different procedures helped me to learn the technical nuances and overcome those difficult technical difficulties

**Table 1:** Identified factors that contribute to the experience in clinical exposure and operating theatre in the EAES fellowship programme

Factors that contribute to experience in clinical & operating theatre exposures	Codes
Supporting Factors	
Positive attitudes of the host	4
Expertise of the host	2
Hands-on participation	2
Opposing Factors	
Language barrier	6
Lack of scrubbing opportunities	2
Lack of non-operative clinical activities	2
Lack of medical registration for scrub in operations	2

On the other hand, the most frequently stated negative factor to the experience was the language barrier (6 codes).

The primary language of communication in ward rounds, group discussions and outpatient clinics was Italian. That made my participation difficult in many clinical activities.

I wish I had taken some lessons as the language barrier did limit my exposure significantly.

For some fellows, hands-on exposure was limited by the lack of scrubbing opportunities commonly due to the lack of medical registration to scrub (2 codes). A few commented on the limited scope to join clinical activities that led to little experience in ward rounds and outpatient clinics.

I think that the problem of scrubbing opportunities should be addressed and improved

…I did not have the chance to be included in ward rounds to observe the outcome of the procedures I saw.

### Research and education exposure

Ninety-one point two percent (91.2%) of fellows felt that they had good access to educational activities such as local teaching and meetings and 23.5% (8.8% strongly agree, 14.7% agree) were able to contribute through teaching. 55.9% had good access to research activities; 44.1% participated in these activities and 41.2% had the opportunity to be an author/co-author of a publication from the host research group.

One single barrier that restricted the exposure to research was duration of the fellowship; this was mentioned seven times in the feedback.

….I expressed my desire to participate in research. However, I could not extend my stay due to my visa.

I was supposed to return to the institution after a few months for further educational and research opportunities, but this was not permitted by my hospital chief due to lack of available surgeons at the moment.

### Impact

Ninety-one point seven percent (91.7%) of fellows stated that their operative exposure and competency had increased as a result of the fellowship (65.7% strongly agreed and 31.4% agreed). 94.3% felt that they have gained new surgical skills and 85.7% was able to introduce the new skills in their own hospitals and clinical practices. 74.29% of fellows agreed that the clinical experience led to a change in their own or hospital practices.

I also got the opportunity to learn many new technical skills, which have helped me to improve my practice and helped in training my residents back home in a better way.

One fellow (2.9%) did not gain any new skills and one (2.9%) was not able to introduce new techniques in his/her hospital. This was due to the limitation of local equipment and access to appropriate clinical cases.

I wanted to start with TATME (Transanal Total Mesorectal Excision), but the problem is/and was lack of instruments.”

Support from the head of department helped a lot but lack of appropriate easy cases to start with is a problem.

The research and educational exposure were thought to be increased in the majority of the fellows (62.8% agreed and strongly agreed). After the fellowship period, 20% of the fellows continued to participate in the educational activities and 31.4% in research in the host institute.

The fellowship has also given me a different perspective in educating residents and colleagues, particularly how accommodating the people were in helping me understand the procedures and

The weekly meetings with the discussion of the pre and post-operative management of complex cases were interesting and useful to increase my knowledge on operative decision making. The opportunity to attend different courses/conference gave me the gimmick to develop new research projects and to interact with several colleagues.

### Improvement

The most commonly suggested improvements include setting realistic target for clinical and research areas (5 codes), increasing the duration of fellowship (4 codes), and increase theatre assisting opportunities (3 codes) (Table 2).

Guaranteeing to all the fellow a full clinical and research fellow, with clear and delineated target

Perhaps, minimum number of surgical procedures for observation or assistance should be standardized for each institution.

longer stay for greater experience.

It is important to the program that every fellow could have some degree of OR exposure.

**Table 2:** Suggested areas of improvement for the EAES fellowship programme

Suggested improvements	Codes
To set realistic target for both clinical/theatre assisting and research areas	5
To increase the duration of the fellowship	4
To increase theatre assisting opportunities	3
To address language barrier issues	1

### Overall

100% of fellows would recommend the EAES fellowship to their peers (94.3% strongly agree, 5.7% agree).

## Discussion

Internationalisation has been key to sharing information, education and dissemination of innovation in the revolution of Surgery [4]. The EAES fellowship programme has enabled the exchange of surgical skills and techniques since 2014. Amongst the 42 fellows between 2014 and 2018, 42.8% were from Central and Eastern Europe, 28.6% from Southern Europe, 9.5% from Western Europe, 2.4% from Northern Europe and 16.7% from non-European countries including Philippines, Tunisia and Panama. Many fellowship programmes including the American College of Surgeons were set up to promote education and collaboration with other countries and their scientific organisations [5]. With a strategy to expand the global network, international guest scholarship (IGS) and community travel awards were designed for surgeons in countries other than USA and Canada or from poorly resourced academic departments in developing countries. This was mutually beneficial for the US surgeons, as through this “window to the world”, they could learn more about practice of surgery at an international level. In parallel, the selection process in EAES fellowship focuses on equal access opportunities to surgeons of all backgrounds and the potential impact that the programme might bring to the individual as well as the home institute. The heterogeneity of the cohort is under constant evaluation with the future plan for expansion.

Hands-on surgical exposure has been a key element of the EAES fellowship programme with good access to operating rooms for all fellows and scrubbing opportunities for more than 70%. Non-operative exposures including other clinical activities, education and research were also seen as essential in offering a holistic experience to surgical care. More than 90% of fellows described gaining new surgical skills as a result of the fellowship, and 85% was able to introduce a change in the practice of their own and that of their hospitals. The free-text feedback showed a positive impact on skills acquisition in dealing with technical difficulties and methods of surgical training.

Addressing language barriers is essential to improving learning from the ward and outpatient environment; this may be overcome by careful selection of hosts according to language abilities, local arrangements of assistance, and setting expectations. Similarly, scrubbing opportunities may be affected by medical licensing regulations which should be considered during the host selection. Conducting research is challenging during a 3-month fellowship window. Collaborating prior to the visit and the fellow’s commitment beyond the fellowship period would be necessary to achieve sustainable relationship. The overall experience could be enriched by establishing realistic and individualised targets in areas of surgical skills, clinical exposures, education and research. Moreover, different types of EAES fellowships may be developed in the future focusing on the exchange of information in various aspects of surgery. For example, in additions to the IGS programme, the ACS also offers Surgical Education scholarship for those who have major interests in surgical training, National Surgical Quality Improvement Programme (NSQIP) scholarship for quality improvement, and Reciprocal International Travelling Fellowship for basic science, and clinical/translational studies [5].

In 2020, EAES launched the Forward Project to address the emerging needs for initiating and supporting the spread of MIS in some European countries and the limitations of a short-term fellowship. It is a 2-year structured longitudinal programme consisting of specialty-specific teaching, which in turn became a webinar programme in response to the Covid-19 pandemic, a 3-month clinical visit in a recognised MIS centre, and proctorship at the fellow’s centre to practice learnt skills. Ten young surgeons from South Europe including Bosnia & Herzegovina, Montenegro, Kosovo, Serbia and Macedonia are currently undertaking the fellowship.

Although the presented data suggest tremendous success of the EAES fellowship programme, care must be taken not to forget the missing data from the 17% of non-responders. Although this may be due to various reasons, one may speculate that refusal to participating in the survey could be due to negative experience rather than the opposite. In additions, fellows may have different needs and expectations according to their stages of career, geographical origins, and economical backgrounds. Therefore, future studies may be designed to extract details on the impact of the fellowship programme from this perspective.

## Conclusion

In its first 5 years, the EAES fellowship programme has made a significant impact on acquiring and adopting new MIS techniques based on the experience of fellows between 2014 and 2019 revealed in this study. To further refine the programme an individualised approach should be adopted. The use of more structured feedback would help to better match fellows’ expectations and help set achievable learning objectives in clinical skills, education and research.

## Supplementary Information

Below is the link to the electronic supplementary material.

Supplementary file1 (PDF 116 kb)
