# Supplementary material for: The impact of EAES Fellowship Programme: a five-year review and evaluation
Source: Surg Endosc. 2021 Jun 8;36(4):2430–5. doi: 10.1007/s00464-021-08525-8 (PMC8186018; doi:10.1007/s00464-021-08525-8)
Supplement: Supplementary file 1 — Supplementary file1 (PDF 116 kb) [file 464_2021_8525_MOESM1_ESM.pdf]

EAES FELLOWSHIP EVALUATIOJN						
**Demographic**						
During the fellowship, I was a	Surgeon (consultant)	How many years of independent practice:____________________________				
	Resident (registrar)	How many years of postgraduate training: ____________________________				
Which specialty(ies) did you specialise in:						
Which host organisation did the fellowship take place in?						
Which specialty(ies) of surgery were you attached to during the fellowship?						
**During the Fellowship Period,**		**Strongly disagree**	**Disagree**	**Neutral**	**Agree**	**Strongly agree**
**Operative & Clinical Experience**						
I had a good access to attending /observing the operating theatres.						
I was able to assist in the operations.						
I had a good access to the clinical activities such as ward rounds and outpatient clinics.						
I was able to participate and contribute to the clinical activities						
Other comments:						
**Education & Research Experience**						
I had a good access to the educational activities such as local teachings and meetings.						
I was able to participate in educational activities.						
I was able to teach in the educational activities.						
I had a good access to the research activities such as studies conducted during the fellowship.						
I was able to participate in the research activities.						
I had the opportunity to be an author/co-author of an article published from the host research group.						
Other comments:						
**After the Fellowship Period ,**		**Strongly disagree**	**Disagree**	**Neutral**	**Agree**	**Strongly agree**

**Operative & Clinical Skills**					
My operative exposure and competency had increased as a result of the fellowship.					
I have gained new surgical skills/techniques during the fellowship.					
I was able to introduce the new skills/techniques in my own hospital/clinical practice					
Please describe factors that made adoption easy or difficult (e.g. support from chief surgeon, lack of instrument, etc…): ____					
My clinical exposure of the specialty had increased as a result of the fellowship					
The clinical experience led to a change in my own hospital / clinical practice					
Other comments:					
**Education & Research Skills**					
I continue to participate in the educational activities in the host organisation.					
I continue to have an academic relationship with the research group in the host organisation.					
My academic and research exposure to the surgical speciality had increased as a result of the fellowship.					
Other comments:					
**OVERALL**	**Strongly disagree**	**Disagree**	**Neutral**	**Agree**	**Strongly agree**
I would recommend the EAES fellowship to my peers.					
Because:					
EAES Fellowship can be improved by:					
